# Characterization and non-parametric modeling of the developing serum proteome during infancy and early childhood

**DOI:** 10.1038/s41598-018-24019-5

**Published:** 2018-04-12

**Authors:** Niina Lietzén, Lu Cheng, Robert Moulder, Heli Siljander, Essi Laajala, Taina Härkönen, Aleksandr Peet, Aki Vehtari, Vallo Tillmann, Mikael Knip, Harri Lähdesmäki, Riitta Lahesmaa

**Affiliations:** 1 Turku Centre for Biotechnology, University of Turku and Åbo Akademi University, Turku, FI-20520 Finland; 2 Department of Computer Science, Aalto University School of Science, Aalto, FI-00076 Finland; 3 Children’s Hospital, University of Helsinki and Helsinki University Hospital, Helsinki, FI-00029 Finland; 4 Research Programs Unit, Diabetes and Obesity, University of Helsinki, Helsinki, FI-00014 Finland; 5 Department of Pediatrics, University of Tartu, 50090 Tartu, Estonia; 6 Children’s Clinic of Tartu University Hospital, 50406 Tartu, Estonia; 7 Helsinki Institute for Information Technology HIIT, Department of Computer Science, Aalto University, Aalto, FI-00076 Finland; 8 Folkhälsan Research Center, Helsinki, FI-00290 Finland; 9 Tampere Center for Child Health Research, Tampere University Hospital, Tampere, FI-33014 Finland

## Abstract

Children develop rapidly during the first years of life, and understanding the sources and associated levels of variation in the serum proteome is important when using serum proteins as markers for childhood diseases. The aim of this study was to establish a reference model for the evolution of a healthy serum proteome during early childhood. Label-free quantitative proteomics analyses were performed for 103 longitudinal serum samples collected from 15 children at birth and between the ages of 3–36 months. A flexible Gaussian process-based probabilistic modelling framework was developed to evaluate the effects of different variables, including age, living environment and individual variation, on the longitudinal expression profiles of 266 reliably identified and quantified serum proteins. Age was the most dominant factor influencing approximately half of the studied proteins, and the most prominent age-associated changes were observed already during the first year of life. High inter-individual variability was also observed for multiple proteins. These data provide important details on the maturing serum proteome during early life, and evaluate how patterns detected in cord blood are conserved in the first years of life. Additionally, our novel modelling approach provides a statistical framework to detect associations between covariates and non-linear time series data.

## Introduction

Serum and plasma are relatively easily and non-invasively collected as clinical samples for diagnostic purposes. Typically, targeted protein measurements of these samples include several key markers that reflect metabolism, inflammation and lipid profiles. In addition to these, this liquid component of the blood also carries a wide representation of the human proteome, ranging from highly abundant carrier proteins, such as albumin, down to low abundance cytokines, which may collectively reflect the individual’s phenotype and health status^1,2^. With current mass spectrometry-based proteomics technologies it has become possible to study the expression levels of hundreds of proteins from only a few microliters of serum or plasma^3^. Such profiling could enable more detailed health monitoring moving closer towards personalized medicine^4^.

A number of recent studies have investigated the sources and levels of variation for human plasma proteins^3,5–7^. High levels of inter-individual variability have been observed for many plasma proteins^1,4^, indicating that there are already differences in plasma proteomes of healthy individuals. On the other hand, a significant degree of intra-individual stability has been detected for many plasma proteins in adults, both within a short interval of a few days^3^ as well as across several years^6^. However, in a recent study by Liu *et al*.^5^, based on longitudinal follow-up samples collected from ten children between the ages of 1–14 years, temporal age-associated expression level changes were reported for approximately half of the quantified serum proteins.

Children develop rapidly during the first few years of their lives, and the major changes during that time are likely to be reflected in biomolecules, such as proteins, circulating in the blood stream. The first years of life are also important for predicting and monitoring certain childhood diseases, such as type 1 diabetes, where the first signs of autoimmunity are often detected already during the first two years of life^8,9^. Prenatal factors have also been associated with increased risks for certain diseases such as asthma^10^, similar to well-characterized genetic risk factors for several diseases^11^. An understanding of the changes in the serum proteome during early life is therefore important when trying to distinguish the differences between healthy and disease states.

Identifying different sources of variability is challenging in longitudinal studies with complex omics data. Traditional methods have used linear mixed models, where nonlinearity is obtained by introducing polynomial terms^5^. However, these standard models require substantial knowledge of the studied phenomenon in order to select appropriate nonlinear effects and to choose covariance structures to model correlated outcomes; such knowledge is rarely available. Use of the standard models is also complicated by the need to handle irregular sampling times and missing values, to account for time-varying covariates, and the general challenge of performing model inference. The Gaussian process (GP) regression model is a probabilistic non-parametric modeling framework widely used to model time series or other data that exhibit unknown non-linear trends. Since GPs can model any non-linear smooth function without prior knowledge of the parametric form of nonlinearity, and are very flexible for the modelling of interactions of given covariates, they are ideal for modelling longitudinal biomedical data.

The aim of this study was to establish a reference model for the evolution of a healthy serum proteome during early childhood. Label-free quantitative proteomics analysis was performed for longitudinal serum/plasma follow-up samples collected from Finnish and Estonian children during the first three years of life, as well as for umbilical cord serum samples of these children. A flexible additive GP regression model was developed to account for complex experimental designs in longitudinal studies. Through shared and interacting kernels, the method explicitly models different influencing factors in the additive GP model, such as age, gender, living environment, sampling season and individual variation on protein expression patterns. Using GP modelling of the longitudinal proteomics data from the follow-up samples we were able to simultaneously evaluate the contribution of the different variables on serum protein expression levels. In order to evaluate how prenatal conditions are reflected in the serum proteome, we show for the first time, how protein abundance patterns observed from the analysis of umbilical cord blood serum samples are correlated with those subsequently collected serum samples.

## Results

**Figure 1:**
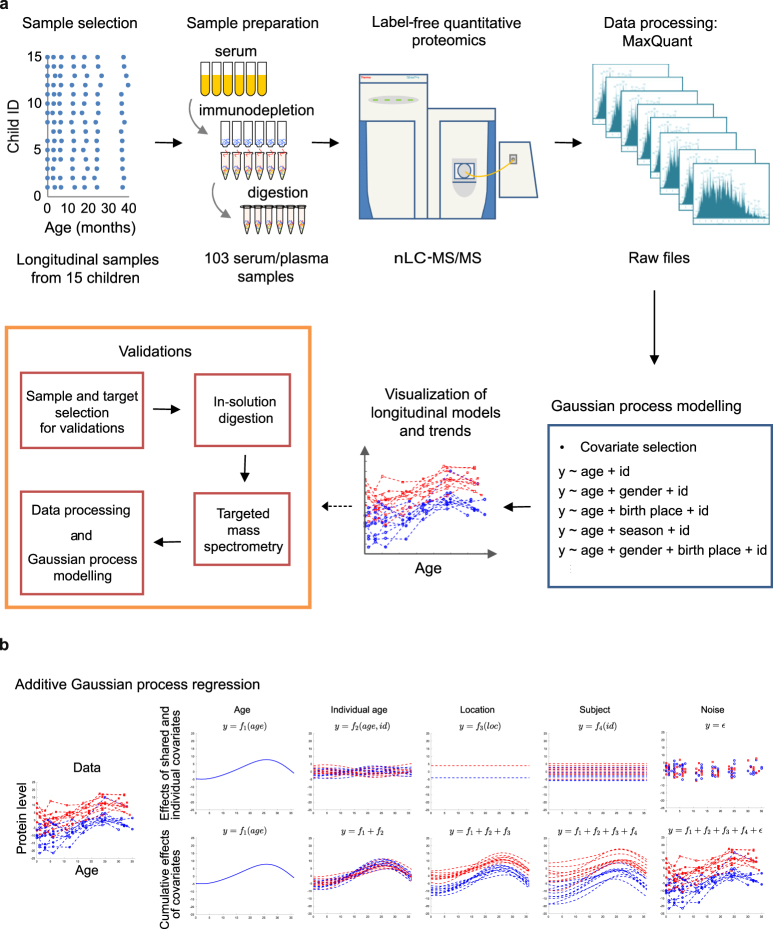
The workflow of the study. (**a**) A schematic outline of the general workflow of the study. (**b**) An illustration of additive GP modeling. Simulated protein data shown on the left (and bottom right) consists of additive effects of individual covariates (age, location, subject ID), their interactions (age and subject ID) and noise shown on the top row. The cumulative effects of these additive components are shown on the bottom row (from left to right). While the effects of different additive terms are shown as continuous functions, the data (and noise) is measured at selected time points that correspond to the sampling time points in our LC-MS/MS proteomics data.

Label-free quantitative proteomics was used to study the evolution of serum proteome during the first three years of life of fifteen children. Longitudinal follow-up serum samples collected from eleven Finnish and four Estonian children at the ages of approximately 3, 6, 12, 18, 24 and 36 months were analyzed and compared with umbilical cord serum samples collected from 14 of these children (Fig. 1a). To minimize the influence of technical variation on the longitudinal protein expression profiles of each individual, the samples for each subject were prepared and analyzed in the same batch. LC-MS/MS analyses of the longitudinal follow-up samples collected between the ages of 3 months and 3 years resulted in the identification and quantitation of 266 proteins in >50% of the samples (Supplementary Table 1). Analysis of the cord serum samples revealed a more extensive range of detectable proteins. Of the 404 proteins detected in the cord serum, 230 were also detected in the longitudinal follow-up samples (Supplementary Table 2), providing new insights in to the relationship between post-natal serum proteins levels and the cord blood.

Additive GP modelling was used to study the effects of age, gender, living environment, sampling season and individual variation on the longitudinal expression patterns of the 266 proteins (Fig. 1a). An example of additive GP is shown in Fig. 1b. The statistical methods are described in more detail in Materials and Methods section.

### Age is the most dominant factor influencing serum proteomes of young children

**Figure 2:**
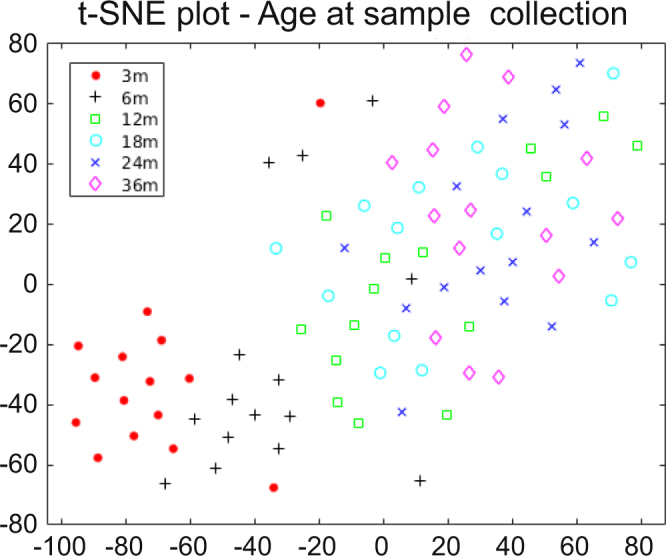
t-SNE plot of all the follow-up samples based on log_2_ intensities of the 266 proteins. The samples have been colored based on the age of the child at sample collection.

t-SNE (t-distributed stochastic neighbor embedding) plots of the longitudinal follow-up samples indicated that age has a strong effect on the serum proteomes of young children (Fig. 2). In particular the samples collected at the ages of three and six months were more distinct from the samples collected at older ages as well as from each other. Based on the t-SNE plots there was no clear separation of the samples based on individual, gender or place of birth (Supplementary Fig. 1).

**Figure 3:**
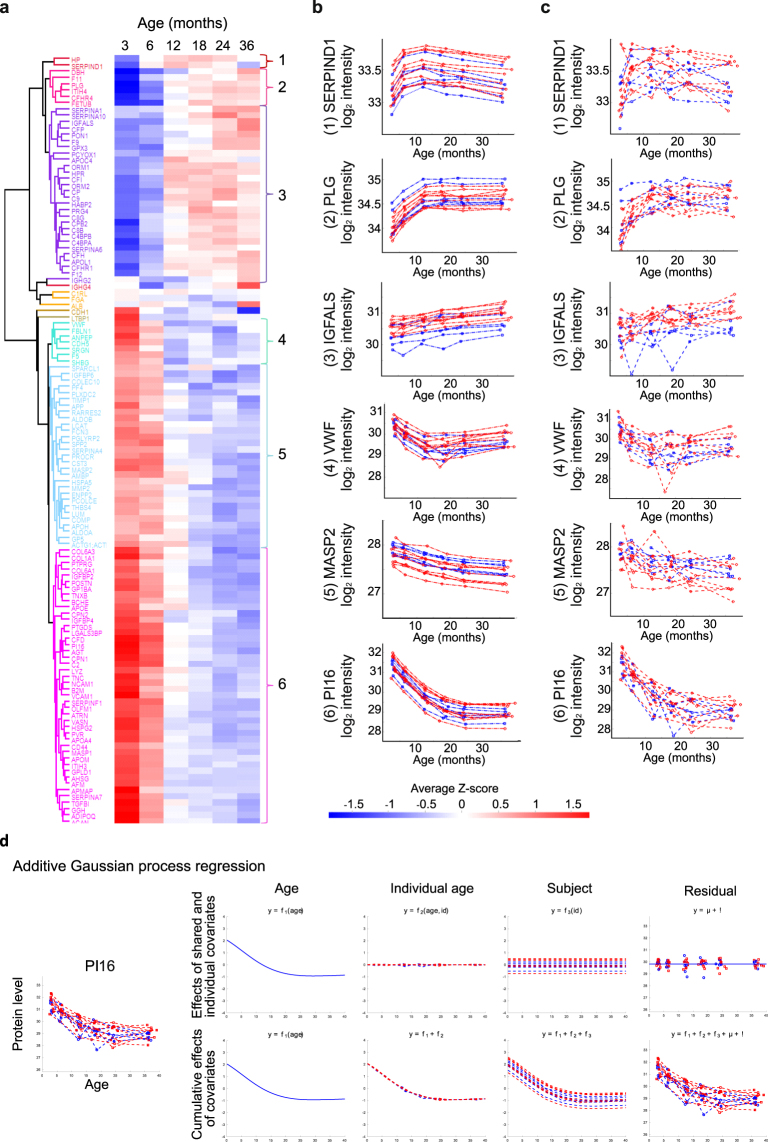
The effect of age on protein expression levels. (**a**) A heatmap visualizing the average age-associated changes of the 122 proteins with age effect. Child-specific Z-scores were first calculated for each protein at all timepoints based on label-free proteomics data, and the averages of these Z-scores across all children were plotted in the heatmap. Example visualizations of (**b**) age-associated GP models and (**c**) corresponding measured protein intensities (in log_2_ scale) for selected proteins present in the six main clusters. Each line represents one child, red = girls, blue = boys. (**d**) Covariate contributions for PI16. Estimated covariate contributions shown on the top row are calculated as the posterior mean predictions of the corresponding components in the age model. The bottom row shows the cumulative contributions these additive components. SERPIND1 = Heparin cofactor 2, PLG = Plasminogen, IGFALS = Insulin-like growth factor-binding protein complex acid labile subunit, VWF = von Willebrand factor, MASP2 = Mannan-binding lectin serine protease 2.

On the basis of GP modelling, 122 proteins displayed significant age-associated changes in their expression levels (Supplementary Table 3). The direction, magnitude and dynamics of the age-associated changes varied clearly between different proteins, but in general the most prominent changes were observed already during the first year of life. A general overview of the age-associated changes in the dataset is presented in Fig. 3a using a heatmap of the average Z-scores for each of the 122 proteins in different age groups. The first three clusters include 35 proteins with temporal increase in their expression. Proteins in clusters 1 and 2 are characterized by clear expression level changes occurring especially at the ages of three and six months, whereas proteins in cluster 3 show more stable temporal increase in expression. Clusters 4–6 include 82 proteins with a decrease in their expression. Of these, proteins in clusters 4 and 6 are characterized by rapid decrease in their expression during the first year of life, whilst in cluster 5 there is a more stable decrease. Examples of the age-associated GP models and measured protein intensities for each of the six clusters are shown in Fig. 3b,c. Finally, there are a few proteins that follow time window-specific changes of expression, including Ig gamma-4 chain C region (IGHG4) with a clear drop in expression at the age of 6 to 12 months. Based on the GP modelling, peptidase inhibitor 16 (PI16) was amongst the proteins with the strongest age-associated changes in expression (Fig. 3d).

Significant temporal changes in the plasma proteome during childhood have been recently described in two studies^5,7^. Two thirds of the proteins with age-associated changes in the current study were also reported to display temporal changes by Liu *et al*.^5^, based on 90 longitudinal follow-up samples collected from ten children across a broader age range of 1–14 years (Supplementary Table 3). However, we also observed temporal changes in 40 proteins for which changes were not detected in the data of Liu *et al*.^5^. These included heparin cofactor 2 (SERPIND1), basement membrane-specific heparan sulfate proteoglycan core protein (HSPG2) and attractin (ATRN), with the biggest age-associated changes observed during the first year of life. More recently, in a cross-sectional study by Bjelosevic *et al*. age-associated differences were studied in the plasma proteomes of three groups of children (infants, children under the age of 1 y and 1–5 y old children) and adults^7^. 31% of the proteins with significant temporal changes in the current study were also reported with age-associated changes in their study (Supplementary Table 3), including, for example, early changes in the expression levels of SERPIND1. In contrast to the current study and the observations of Liu and co-workers, temporal expression level changes were reported for less than 15% of the total 940 proteins quantified. However, it should be noted that this result was based on the use of an extensive third party SWATH-MS spectral library. When a local spectral library of 151 proteins was used, temporal changes were observed for approximately 50% of these proteins, indicating that the lower total percentage of temporally changing proteins could be due to increased noise with the larger spectral library^7^.

To further confirm age-associated changes, targeted single reaction monitoring (SRM) mass spectrometry analysis was performed for three selected proteins in 39 samples collected from eight additional children from the same study cohort. GP modelling of the SRM data for coagulation factor IX (F9), poliovirus receptor (PVR) and transforming growth factor-beta-induced protein ig-h3 (TGFBI) confirmed significant age-associated expression level changes for these proteins (Supplementary Fig. 2).

### Functional roles of the proteins with age-associated expression level changes

**Figure 4:**
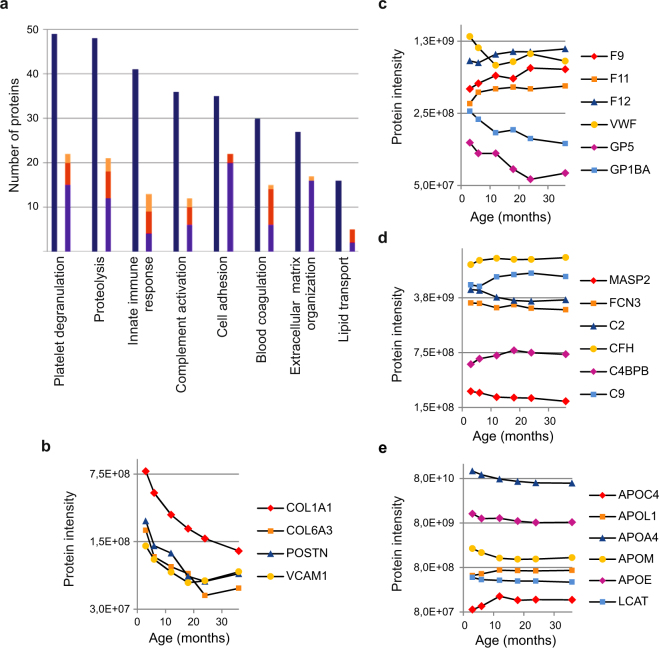
Functional classification of the proteins. (**a**) Numbers of proteins mapping to different Gene Ontology classes. Dark blue = all quantified proteins, blue = proteins decreasing with age, red = proteins increasing with age, yellow = proteins with timepoint-specific temporal changes. Examples of temporal changes in (**b**) cell adhesion and extracellular matrix-associated proteins, (**c**) proteins involved in blood coagulation, (**d**) complement proteins and (**e**) apolipoproteins based on median protein intensities in each age group are shown. COL1A1 = Collagen alpha-1(I) chain, COL6A3 = Collagen alpha-3(VI) chain.

Functional classification of the proteins quantified in this study was performed using DAVID bioinformatics tool^12^. Using the whole human proteome as the background the top 20 most significantly enriched biological processes covered several functional classes typical for serum proteome (Fig. 4a, Supplementary Table 4). However, when functional enrichment analysis was performed for the 122 proteins with age-associated changes using only the reliably quantified proteins as background, no biological processes were significantly enriched. This indicates that the age-associated changes during early life are observed across a range of different functional categories of serum proteins.

Temporal changes for proteins associated with cell adhesion and extracellular matrix organization were almost exclusively characterized by age-associated decrease (Fig. 4a). Similarly, cell adhesion-associated proteins were enriched among the proteins with a temporal decrease in expression levels in the study by Liu *et al*.^5^. In particular, collagens, the main structural proteins in extracellular matrix, showed a pronounced decrease in expression levels during the first three years of life (Fig. 4b). Amongst the interesting examples with a clear age associated decrease in their serum expression levels were periostin (POSTN), which is involved in cell attachment and is thought to be a potential biomarker for pediatric asthma^13^, and vascular cell adhesion protein 1 (VCAM1), which is involved in leukocyte-endothelial cell adhesion.

Blood hemostasis is known to differ between young children and adults, and accordingly changes in the expression levels of several proteins involved in blood coagulation have been observed especially during the first year of life^14,15^. We observed an age-associated increase in the expression levels of several hemostasis-associated proteins, including coagulation factors IX (F9), XI (F11) and XII (F12) (Fig. 4c). On the other hand, an age-associated decrease was detected in the levels of von Willebrand factor (VWF), which promotes platelet adhesion to the sites of vascular injury, platelet glycoprotein V (GP5), a mediator of this process, and platelet glycoprotein Ib alpha chain (GP1BA), which is also involved in the formation of platelet plugs via binding to VWF (Fig. 4c).

The complement system, a key component of innate immune response, is known to be underdeveloped in newborn infants and several studies have detected lower levels of most complement proteins in healthy newborns compared to healthy adults, as reviewed by McGreal *et al*.^16^. Additionally, a number of studies have looked into the age-associated changes in complement protein expression levels during childhood and observed temporal increase in the levels of many complement proteins^5,17,18^. In the present study temporal increase was observed in the levels of Complement factor H (CFH), complement factor I (CFI) and C4b-binding protein alpha and beta chains (C4BPA, C4BPB), which act as negative regulators of complement pathway, and the levels of complement factor H-related protein 1 and 4 (CFHR1, CFHR4) that also regulate complement activity (Fig. 4d). The same trend was observed for several components of the membrane attack complex, i.e. complement component C8 beta and gamma chains (C8B, C8G) and complement component C9 (C9). Interestingly, we also observed age-associated decrease in the expression levels of five complement proteins involved in early stages of complement cascade activation, i.e. mannan-binding lectin serine protease 1 and 2 (MASP1, MASP2), ficolin-3 (FCN3), complement component C2 (C2) and complement factor D (CFD) (Fig. 4d).

In addition to complement proteins, several other proteins of the immune system were also quantified. For many of these, including interleukin-1 receptor accessory protein (IL1RAP) and neutrophil defensin 3 (DEFA3), we did not observe any consistent age-associated expression level changes during early life. However, two IgG components, Ig gamma-2 chain C region (IGHG2) and IGHG4, were seen to decrease in their expression levels until the age of six months, followed by an increase after the first year of life. This is supported by age-specific IgG reference values in healthy babies from earlier literature^19^, and is in line with the idea that newborn infants with an immature immune system are protected by maternal IgG transported transplacentally during pregnancy, before the infant’s own IgG production is established^20^.

The GP models indicated age related trends for six out of the 15 different apolipoproteins quantified in this study. The levels of apolipoproteins APOC4 and APOL1 were increasing and the levels of APOA4, APOM, APOE and APOH were decreasing with age (Fig. 4e). A temporal decrease was also observed in the levels of phosphatidylcholine-sterol acyltransferase (LCAT), an important enzyme in extracellular metabolism of plasma lipoproteins. Most of the proteins associated with proteolysis or platelet degranulation did not show age-associated changes in their expression profiles.

### Individual variation in the serum proteomes

In addition to age, individual variability is likely to affect plasma protein expression levels in small children. Since the current study was designed to minimize technical variation in longitudinal expression profiles of individual children, longitudinal serum samples collected from 1–2 children were prepared and analyzed in the same batch. Consequently, the subject ID covariate in the GP models enables accounting for the individual variation that reflects both true individual variation and possible technical batch-to-batch variation. Therefore, the significance of individual variation on protein expression levels was additionally evaluated on the basis of four pairs of children, whose samples were prepared and analyzed in the same batches (Supplementary Table 5).

For 67 of the 122 proteins with a significant age effect, the contribution of individual variation on the best GP model of protein expression was higher than the contribution of age-associated variation in terms of variances of the corresponding GP components (Supplementary Table 5). When studying the paired children, the median fold-changes across all time points were ≥1.5 for 23 of these proteins. The contribution of individual variation was higher than the contribution of noise for further 41 proteins with no significant age effect. Here the median fold-changes for paired children across all time points were ≥1.5 for 17 of the proteins.

**Figure 5:**
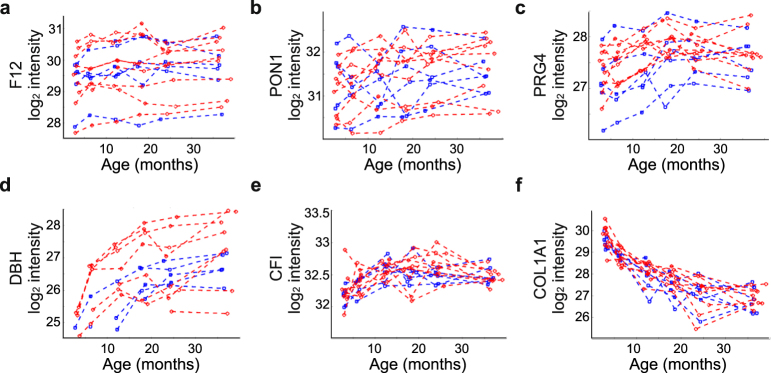
Individual-specific variation in protein expression levels. Different levels of individual variation were observed for (**a**) F12, (**b**) PON1, (**c**) PRG4, (**d**) DBH, (**e**) CFI and (**f**) COL1A1. Each line represents one child. Red = girls and blue = boys.

Amongst the proteins with large individual variation were several proteins with known SNPs, which have previously been observed to influence protein abundance^6,21^, including F12 and serum paraoxonase/arylesterase 1 (PON1) (Fig. 5a,b). High inter-individual variation was also observed e.g. for proteoglycan 4 (PRG4) and dopamine beta-hydroxylase (DBH) (Fig. 5c,d). On the other hand, GP modelling showed that individual variation contributed only slightly to the expression levels of some proteins, for example, complement factor I (CFI) and collagen COL1A1 (Fig. 5e,f).

### The effects of gender and living environment on serum protein expression levels

The effects of gender, birth place and seasonal variation on serum protein expression levels were also studied using GP modelling (Supplemental Table 6). The combination of age, gender and individual variation best explained the longitudinal protein expression patterns for three proteins: pregnancy zone protein (PZP), insulin-like growth factor-binding protein complex acid labile subunit (IGFALS) and carboxypeptidase B2 (CPB2), whereas the longitudinal expression profiles of apolipoprotein C-III (APOC3) were best explained by the combined effects of age, gender, birth place and individual variation. Higher levels of PZP and IGFALS were observed in girls, whereas the levels of CPB2 were higher in boys.

GP was also used to evaluate the influence of sampling season on serum protein expression levels. Sampling season appeared to have a significant contribution on the expression levels of only one protein, although for this there were several missing values in the data. The difficulty in detecting protein expression level changes associated with seasonal variation in this study might also be due to the strong age effect during the first year of life combined with sparse sampling at later time points.

**Figure 6:**
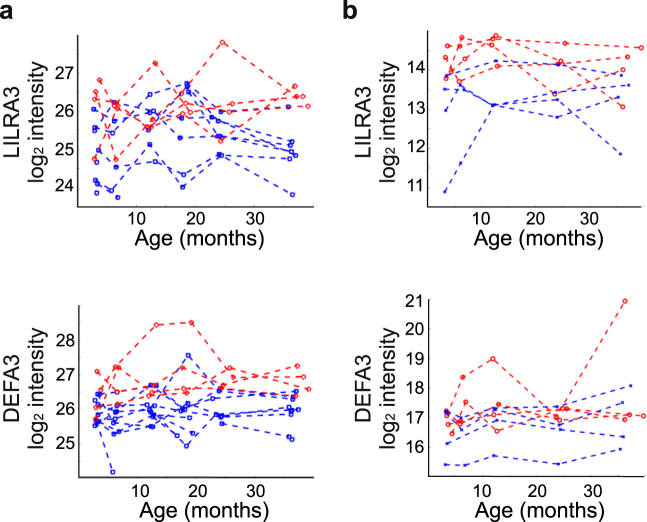
The effect of living environment on protein expression levels. Levels of LILRA3 and DEFA3 based on (**a**) label-free quantitative proteomic profiling of longitudinal serum samples from 15 children (**b**) targeted SRM-based analysis of follow-up samples from 8 additional children. Red lines = Estonian children, Blue lines = Finnish children.

On the basis of the GP modelling, the place of birth had a significant contribution on the expression levels of seven proteins (Supplemental Table 6). In addition, the place of birth had a suggestive contribution on the expression levels of further 17 proteins (Supplemental Table 6). Out of those with higher expression levels in Estonian than Finnish children, six proteins (DEFA3, F9, Fibronectin (FN1), Leukocyte immunoglobulin-like receptor subfamily A member 3 (LILRA3), PVR and TGFBI) were selected for further validation. Targeted SRM analyses were performed for a set of 39 serum samples collected from additional four Finnish and four Estonian children from the same study cohort. These data also indicated higher levels of the two immune-related proteins, LILRA3 and DEFA3 in the Estonian children (Fig. 6). For these proteins the place of birth was included in the best prediction models also in the validation data, but the models did not reach the same significance criteria used with the original data, possibly partly due to the smaller sample number in the validation data. No difference was observed for the other four proteins in the validation experiments.

### Comparison of cord serum proteomes with the follow-up samples

Finally, umbilical cord serum proteomes were analyzed to study whether prenatal conditions are reflected in the serum proteome during early life. In total 404 proteins were identified and quantified in at least 50% of the cord blood samples. Only 230 of these proteins were consistently quantified in the follow-up serum samples (Supplementary Table 2), indicating that umbilical cord serum differs significantly from serum samples collected only a few months after birth. Therefore, these two datasets were processed separately and their subsequent cross comparison was performed based on ranked intensities.

**Figure 7:**
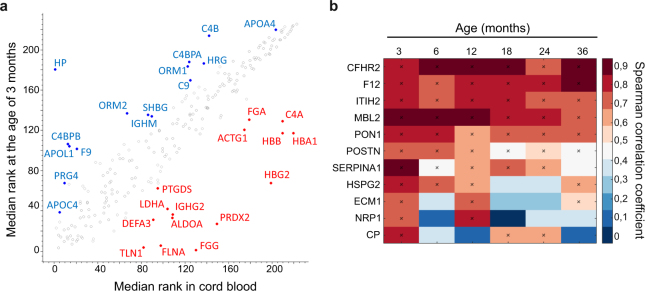
Comparison between the umbilical cord blood and the follow-up samples. (**a**) Median intensity-based ranks between the umbilical cord blood and the 3-month serum samples. The top 15 proteins having the highest and the lowest fold changes (rank product pfp < 0.05) between the two sample types are highlighted with red and blue. Red = protein relative expression level higher in cord blood samples, blue = protein relative expression level higher in 3-month samples. (**b**) A heatmap showing proteins with significant correlations between the umbilical cord blood and the follow-up samples of the same child. Statistically significant correlations (FDR < 0.1) are marked with a cross.

Most of the proteins quantified in both cord serum and follow-up samples had similar intensity-based ranking in the two sample types (Fig. 7a). However, on the basis rank product analysis, 35 proteins had significantly higher relative expression levels in cord blood than their 3-month samples, half of which were shown by GP modelling to also display an age-associated decrease during early childhood. The relative abundances of hemoglobins were clearly higher in cord sera relative to the serum samples collected at the age of three months. After birth the ranks of hemoglobins HBA1 and HBB remained fairly constant, while the ranks of hemoglobin subunit gamma-2 (HBG2), which is part of fetal hemoglobin, decreased clearly with age. A significantly higher relative expression of peroxiredoxin-2 (PRDX2) was also observed in cord blood, potentially reflecting an increased need for antioxidants during the transition from hypoxic intrauterine conditions to a normoxic environment at birth^22^. On the other hand, 41 proteins had significantly lower relative intensities in cord blood when compared with 3-month samples, and an age-associated increase in the expression levels for 44% of these proteins was detected during the follow-up. Several complement proteins were included in this group, supporting the concept of an immature complement system in newborn infants^16^. Also, the relative expression levels of multiple apolipoproteins were significantly lower in cord serum when compared with 3-month serum samples.

Correlations between protein intensities in the cord blood and the subsequent follow-up samples from each individual revealed that the expression levels for eleven proteins were significantly correlated with the initial levels (Fig. 7b). The relative expression levels of Complement factor H-related protein 2 (CFHR2), F12, Inter-alpha-trypsin inhibitor heavy chain H2 (ITIH2), Mannose-binding protein C (MBL2), PON1 and POSTN in the follow-up samples showed significant correlations with cord blood throughout the 3-year follow-up. In addition, the relative expression levels of Alpha-1-antitrypsin (SERPINA1), HSPG2, Extracellular matrix protein 1 (ECM1), Neuropilin-1 (NRP1) and Ceruloplasmin (CP) in cord blood correlated significantly especially with the earliest follow-up samples.

## Discussion

An understanding of the sources and magnitudes of variation of human serum proteins in different populations is a fundamental consideration for serum based diagnostics and biomarker discovery^1,2^. In this study we have evaluated the effects of age, individual variation, gender, living environment and sampling season on serum protein expression in infants and young children based on longitudinal serum proteomics analyses of umbilical cord blood samples and six follow-up samples collected from 15 children during the first three years of life.

Longitudinal biomedical data can often exhibit non-linear trends, and there is usually insufficient prior knowledge on how to parameterize the shape of such unknown trends. As GP places no parametric restrictions on the underlying function except for the smoothness, it is a useful tool to model this type of data. GP can also easily capture characteristics and dependencies specific for certain applications and experimental designs, such as minor individual-specific deviations from the average pattern shared by all individuals. This kind of hierarchical modeling allows us to reasonably place variances across different covariates and their interactions. The results enable us to separate the contribution of each component, as well as joint contribution of several components. Moreover, as we show in another manuscript [https://www.biorxiv.org/content/early/2018/02/06/259564], the additive GP model together with Bayesian inference and model selection method is accurate in detecting covariate effects even with small sample sizes.

On the basis of GP modelling of the serum proteomics data, age was the most dominant factor influencing the levels of almost 50% of the quantified proteins in young children. The significance of age-associated changes in the levels of circulating proteins during childhood has already been recognized with the need for age-specific reference values for several clinical assays^14,15,18,23^. Notably, 18 of the 38 FDA-approved proteins measured in serum or plasma^24^ and quantified in the current study showed age-associated expression level changes based on GP modelling. The opportunities and suitability of global mass spectrometry-based proteomics approaches for a broader understanding of the normal development of serum or plasma proteome have already been recognized^5,7,25^. Liu *et al*.^5^ have evaluated temporal changes in the plasma proteome over a broad age range of 1–14 years, and many of the observations in the current study are also reflected in their data. Despite the lower coverage of serum proteome in our study, our data notably compares for the first time the proteomes of umbilical cord sera and subsequent samples from the same individuals, defining in detail the early changes during the first 36 months. We identified temporal changes in 40 proteins not recognized in the previous longitudinal study by Liu *et al*.^5^. Many of these proteins displayed rapid changes in their expression during the first year of life, indicating differences in the temporal changes occurring in serum/plasma proteome at different times during childhood and thereby highlighting the importance of detailed characterization of temporal changes in age-windows of interest. Some of the early changes observed in the current study were also reported in the cross sectional studies by Bjelosevic *et al*.^7^ and Ignjatovic *et al*.^25^ investigating changes in plasma proteomes from neonates to adults.

In the current study, clear differences in serum protein expression levels were observed especially between the first six months and the later ages. The first year of a child’s life is characterized by significant changes in the body, nutrition and living environment and accompanied with rapid growth and development. During the first few months infants get their nutrition from breastmilk or formula, and introduction of solid foods is often initiated around the age of 6 months. In the first year of life children also learn to crawl or walk around independently and are exposed to larger variety of substances in their environment. A number of significant temporal changes observed in the current study are likely reflecting these nutritional and developmental changes in addition to the education of the immune system during the first year of life.

High inter-individual variability for several serum proteins has been observed in earlier proteomic studies^1,3,6,26^, and studies on the heritability of the human plasma proteome have also identified several SNPs influencing protein expression levels in plasma^6,21^. Considerable individual differences were also observed for tens of proteins in the current study. Despite high inter-individual variations in the expression levels of these proteins, through the application of GP modelling we were able to extract clear temporal expression patterns for many of such proteins, including F12 and PRG4.

Gender, living environment and seasonal variation could also affect protein expression levels in healthy young children. On the basis of GP modelling of our data, we did not observe any effect of seasonal variation on protein expression levels, and the effect of gender was only observed upon the expression levels of a few proteins. We also compared protein expression profiles between the Finnish children born in Espoo, the second largest city in the country, and Estonian children born in rapidly developing city of Tartu. The levels of two immune-related proteins, LILRA3 and DEFA3, were higher in Tartu in both the original dataset and in the validation data, although this difference was not significant in the validation data on the basis of GP modelling. A larger cohort of children would be needed to confirm this observation and to establish other potential differences in the evolution of serum proteome between children living in different environments.

Education of the immune system is initiated already *in utero*, and prenatal conditions can be associated with increased risks for certain diseases^27^. Many of the proteins that were quantified consistently in both cord blood and follow-up samples had similar intensity-based ranking in the two sample types. Significant correlations were observed for eleven of these proteins between the protein intensities in cord blood and follow-up samples of the same individual. Also, on the basis of GP modelling, almost half of the proteins with significantly different relative expression levels between cord blood and 3-month samples continued to change in the same direction postnatally, indicating that the serum proteome is slowly maturing and adapting to the new living conditions after birth. Overall, our data indicated that protein expression levels in umbilical cord blood were associated with the levels of these proteins in serum at least shortly after birth, implying that fetal conditions might have an important role in the early development of healthy serum proteome.

Collectively our data provide important details on the maturing serum proteome and the temporal changes associated with the first three years of life, including consideration of the influence of prenatal factors on the early development of serum proteome. In addition, our novel modelling approach provides a statistical framework to detect associations between covariates and non-linear time series data, an inference task that is challenging to accomplish with the standard regression methods. This study extends and complements recent work presenting the maturing proteome^5,7,25^ and will provide an excellent basis for further large-scale longitudinal studies to define the concept of healthy serum proteome and the factors influencing it during early childhood. These data and the GP modelling approach will be useful in planning future studies aimed at understanding the development of type 1 diabetes and other pediatric diseases, where the first signs of disease might be detected already at very young age, but can be difficult to identify due to other more pronounced changes occurring in the body. While we do not explicitly address the prediction of disease-associated protein signals in the current study, the age-associated protein profiles identified in our study as well as in the study by Liu *et al*.^5^ provide the necessary baseline of normal proteome development against which any disease-associated deviations must be compared.

## Experimental Procedures

### Sample selection

The serum/plasma samples analyzed in this work were collected as part of the international DIABIMMUNE study. The children selected for this work were born between September 2008 and February 2010 in Espoo, Finland (n = 11) or Tartu, Estonia (n = 4), had HLA-conferred risk for type 1 diabetes but had remained negative for type 1 diabetes autoantibodies throughout the follow-up (Supplementary Table 7). In total 88 serum samples and 1 plasma sample collected approximately at the age of 3, 6, 12, 18, 24 and 36 months were analyzed together with umbilical cord serum samples for 14 of the children available for the current study. The 6-month serum sample for child 3 was excluded from the analyses due to a technical error in sample preparation. Harmonized protocols for the collection and storage of the serum and plasma samples were used at the study clinics. The study followed the guidelines of the Declaration of Helsinki for research on human participants, and the study protocols were approved by the ethical committees of the participating hospitals (the Ethics Committee for gynecology and obstetrics, pediatrics and psychiatry, Helsinki University Hospital, Helsinki, Finland, and the Ethics Committee, Tartu University Hospital, Tartu, Estonia). The parents gave their written informed consent.

### Proteomic sample preparation

All samples from one individual were prepared and analyzed in the same batch, and sample preparation was performed in a blinded fashion. 8 μl of serum was depleted of the most abundant serum proteins using disposable affinity-based Pierce top 12 abundant protein depletion spin columns (Thermo Scientific) according to the manufacturer’s instructions. It has been previously shown that these depletion cartridges provide efficient and reproducible depletion of the targeted serum proteins^28^. Protein precipitation was performed for the depleted samples with the addition of ice-cold acetone 4x the volume of the sample and by storing the mixture at −20 °C overnight. The precipitate was dissolved in 150 μl of 8 M urea in 50 mM ammonium bicarbonate. Disulfide bonds between cysteine residues were reduced with DTT and alkylated with iodoacetamide. The urea concentration was adjusted to 1.4 M by adding 50 mM ammonium bicarbonate, and trypsin was added to digest the proteins into peptides at 37 °C overnight. The samples were desalted with Sep-Pak C18 cartridges (50 mg, Waters) and dried before storing them at −20 °C. Prior to MS analyses the dried samples were re-dissolved into 2% FA 2% ACN and protein concentrations were measured using a NanoDrop-1000 UV spectrophotometer (Thermo Scientific). Approximately 200 ng of each sample was loaded for LC-MS/MS analysis, and the samples were analyzed with LC-MS/MS in triplicate. All the samples in each batch were randomized and analysed with LC-MS/MS once, followed by new randomizations and analyses for the second and third replicates. In order to monitor chromatographic reproducibility across the runs, each sample was spiked in with an aliquote of an iRT standard peptide mixture (Biognosys) (approximately 0.4 μl/injection).

### LC-MS/MS analysis

LC-MS/MS analyses were conducted using an Easy-nLC 1000 liquid chromatograph (Thermo Scientific) coupled to a Q Exactive™ Hybrid Quadrupole-Orbitrap Mass Spectrometer (Thermo Scientific). The peptides were first loaded on a pre-column (0.1 × 20 mm), followed by separation in an analytical column (75 μm x 150 mm), both packed with 5 μm Magic C18 silica particles (Michrom). A binary solvent system consisting of water/acetonitrile (98:2) with 0.2% formic acid (solvent A) and acetonitrile/water (95:5) with 0.2% formic acid (solvent B) was used to separate the peptides during the 90 min analyses (2% to 35% B in 65 min, 35% to 100% B in 15 min, 100% B for 10 min) at a flow rate of 300 nl/min.

MS/MS data were acquired in positive ionization mode using data-dependent acquisition. The MS survey scans were aquired with a resolution of 70 000 across the range of 300–2000 m/z, an AGC target of 10^6^ and a maximum fill time of 120 ms. Up to 10 of the most intense ions with charge >+1 were selected for HCD fragmentation using isolation window of 2.0 m/z and with AGC target of 5 × 10^4^ and a maximum fill time of 240 ms. MS/MS spectra were recorded with 17 500 resolution, and dynamic exclusion window of 20 s was used. The raw mass spectrometry data have been deposited to the ProteomeXchange Consortium via the PRIDE^29^ partner repository with the dataset identifier PXD006775.

### Proteomic data processing

Raw files obtained from the LC-MS/MS analyses were processed using MaxQuant software^30^ version 1.5.2.8 and searched against a SwissProt human protein database (release 04/28/2014, 20 226 sequences) with added iRT peptide sequences and common contaminants using the built-in Andromeda search engine. Trypsin digestion with a maximum of 2 missed cleavages, cysteine carbamidomethylation as a fixed modification, and methionine oxidation as variable modification were selected as the parameters of these searches. “Match between runs” option in MaxQuant was used with matching time window of 0,7 min and alignment time window of 20 min. For the longitudinal samples, on average 31 protein identifications per run were retrieved via matching, and on average each protein was identified by MS/MS in 87% of the runs. The Peptide level false discovery rate (FDR) was set to 1%, and was determined by searching against a concatenated normal and reversed sequence database. Label-free quantitation was performed using the fast LFQ algorithm. Otherwise the default settings in MaxQuant were used in data processing. Protein-level FDR was calculated manually based on the search results and^31^ and was found to be 1,5%.

The database search results with LFQ intensities were analyzed using Perseus^32^. Firstly, reversed hits and proteins identified only by modification site were removed. Proteins identified with <2 unique peptides or proteins quantified in <25% of the runs were filtered out. The Protein intensities in each sample were calculated as the medians of non-zero values of the three technical replicates, and after this additional filtering was performed to remove proteins quantified in <50% of the samples. The missing values in the proteomics data were not imputed. All the data from the follow-up samples from children at the age of 3 months to 3 years were processed together, and the data from the umbilical cord serum samples of the same children was processed as a separate batch.

All the samples were prepared and analyzed with standardized protocols, and the instrument performance was monitored using and in house standard with additional chromatographic evaluation made with synthetic iRT peptides. Based on MaxQuant alignment of all LC-MS/MS runs for the 89 follow-up samples, the maximum difference between the measured and calibrated retention times for the synthetic standard peptides added to each sample was 4 minutes, indicating good chromatographic reproducibility. Also, 137 out of 138 proteins quantified in all 267 follow-up sample runs had an average RSD < 16% for the normalized protein intensities across technical replicates. In addition, we have earlier tested the reproducibility of the immunodepletion cartridges used in the current study by depleting 6 aliquots of a serum sample in parallel, followed by in-solution digestion and LC-MS/MS analysis of the samples (data not shown). This test resulted in an average RSD of 22% for normalized protein intensities of 192 proteins identified and quantified in all 6 replicate analyses of a serum sample.

Rank-based approaches were used to compare data collected from the umbilical cord blood samples and the follow-up samples. Differences in relative paired protein expression levels between umbilical cord blood and 3-month samples were defined using rank product analyses^33^ and were considered significant when percentage of false prediction (pfp) was <0.05. Spearman rank correlations of the individual umbilical cord blood and follow-up samples were considered significant with Benjamini-Hochberg corrected p-value < 0.1.

### Modeling of longitudinal protein expression profiles

Gaussian process regression was used to model the longitudinal changes of each protein. Five covariates (age, season, gender, location and id) were considered to explain the longitudinal changes. In total there are 2^4^ = 16 different combinations of the covariates, each of which represents a different Gaussian process regression model. Cross validation techniques were used to compare the models, the results of which signify the most important covariates that explain the change of a certain protein. In general, if the best model for a protein contains a covariate (i.e. age, season, gender and location), the protein has effect associated with the covariate, e.g. age effect. Leave one out cross validation (LOOCV) was used to detect age/seasonal effect and stratified cross validation (SCV) was used to detect gender/location effects. Empirical criteria were then adopted to select proteins with significant effects. See detailed description in Supplementary Methods 1.

### t-SNE plot

The default setting of the MATLAB tSNE implementation in https://lvdmaaten.github.io/tsne/ was used to generate the t-SNE figures.

### Functional classification of proteins

Functional classification of the proteins was performed using DAVID functional annotation tool^12^. Gene Ontology classes with FDR < 0.05 were considered to be significantly enriched.

### Sample preparation and analysis for targeted validations

For targeted validation experiments, 39 follow-up serum samples collected from additional four Finnish and four Estonian children participating in the DIABIMMUNE study (Supplementary Table 7). Similarly as with the discovery samples, the samples were collected from each child approximately at the age of 3, 6, 12, 24 and 36 months. The target proteins for validation were selected based on GP modelling results of label-free quantitative proteomic profiling data. One to three peptides for each protein were selected as SRM targets based on their uniqueness to the selected protein and their intensities in the original serum proteome profiling data (Supplementary Table 8). The most intense transitions for each peptide were selected for monitoring based on MS/MS spectra of heavy labelled standards of the target peptides.

The samples were prepared and analyzed as one batch in a blinded and randomized fashion. Starting with an initial volume of 2 μl of serum dissolved into 100 μl of 8 M urea, in-solution digestion was performed similarly to the original sample set. The samples were desalted using Sep-Pak C18 cartridges (96-well plate format, 100 mg sorbent per well, Waters) and dried before storing them at −20 °C. Prior to MS analyses the dried samples were re-dissolved into 2% FA 2% ACN and protein concentrations were measured using a NanoDrop-1000 UV spectrophotometer (Thermo Scientific). The samples were spiked in with a mixture of heavy labelled target peptides (purchased from Thermo Scientific) and iRT peptides (Biognosys), and approximately 250 ng of each sample was loaded for targeted LC-MS/MS analysis.

Targeted LC-MS/MS analyses were conducted using Easy-nLC 1000 liquid chromatograph (Thermo Scientific) coupled to a TSQ Vantage Triple Quadrupole Mass Spectrometer (Thermo Scientific). The peptides were separated with a similar system as described above using a 62 min gradient (2% to 45% B in 50 min, 45% to 100% B in 2 min, 100% B for 10 min) at a flow rate of 300 nl/min. The raw SRM data have been deposited to the ProteomeXchange Consortium via the PASSEL^34^ partner repository with the dataset identifier PASS01073.

Raw SRM data was imported to Skyline and the SRM chromatograms were manually inspected for appropriate peak integration. Peak area values were exported and the total intensity of each protein was calculated as the sum of all transition intensities for that protein. Target protein intensities were normalized against the intensities of endogenous alpha-1B-glycoprotein (A1BG) in each sample (Supplementary Table 8). A1BG was selected as a reference protein since it was the third most stable protein in the current label-free quantitative proteomics data (CV% = 13,7 across the longitudinal follow-up samples), and it has also been among the most stable serum proteins in other published^35^ and unpublished data from our group analyzing serum samples from children with similar age range. In addition, of the three stable serum proteins tested, A1BG had the best performance in the SRM analyses.

### Data availability

The raw mass spectrometry data have been deposited to the ProteomeXchange Consortium via the PRIDE^29^ and PASSEL^34^ partner repositories with the dataset identifiers PXD006775 and PASS01073, respectively.

## Electronic supplementary material

Supplementary Data

Supplementary Table 1

Supplementary Table 2

Supplementary Table 3

Supplementary Table 5

Supplementary Table 8
