# Supplementary material for: Characterization and non-parametric modeling of the developing serum proteome during infancy and early childhood
Source: Sci Rep. 2018 Apr 12;8:5883. doi: 10.1038/s41598-018-24019-5 (PMC5897447; doi:10.1038/s41598-018-24019-5)
Supplement: Supplementary file 1 — Supplementary Data [file 41598_2018_24019_MOESM1_ESM.pdf]

SUPPLEMENTARY DATA

Characterization and non-parametric modeling of the developing serum proteome during infancy and early childhood

Niina Lietzén[1*], Lu Cheng[2*], Robert Moulder[1], Heli Siljander[3, 4], Essi Laajala[1, 2], Taina Härkönen[3, 4], Aleksandr Peet[5, 6], Aki Vehtari[7], Vallo Tillmann[5, 6], Mikael Knip[3, 4, 8, 9 †], Harri Lähdesmäki[1, 2 †], Riitta Lahesmaa[1 †]

[1] Turku Centre for Biotechnology, University of Turku and Åbo Akademi University, Turku, FI-20520, Finland

[2] Department of Computer Science, Aalto University School of Science, Aalto, FI-00076, Finland

[3] Children's Hospital, University of Helsinki and Helsinki University Hospital, Helsinki, FI-00029, Finland

[4] Research Programs Unit, Diabetes and Obesity, University of Helsinki, Helsinki, FI-00014, Finland

[5] Department of Pediatrics, University of Tartu, 50090 Tartu, Estonia

[6] Children´s Clinic of Tartu University Hospital, 50406 Tartu, Estonia

[7] Helsinki Institute for Information Technology HIIT, Department of Computer Science, Aalto University, Aalto, FI-00076, Finland

[8] Folkhälsan Research Center, Helsinki, FI-00290, Finland

[9] Tampere Center for Child Health Research, Tampere University Hospital, Tampere, FI-33014, Finland

[*, †] These authors contributed equally to this work

Supplemenary Figure 1.

Supplemenary Figure 2.

Supplementary Table 4.

Supplementary Table 6.

Supplementary Table 7.

Supplementary Methods 1.

Supplementary Tables 1, 2, 3 and 5 are given as separate Excel files.

Supplementary Figure 1. t-SNE plots of all the follow-up samples based on log_2 intensities of the 266 proteins. The samples have been colored based on A) individual B) gender and C) place of birth.

A

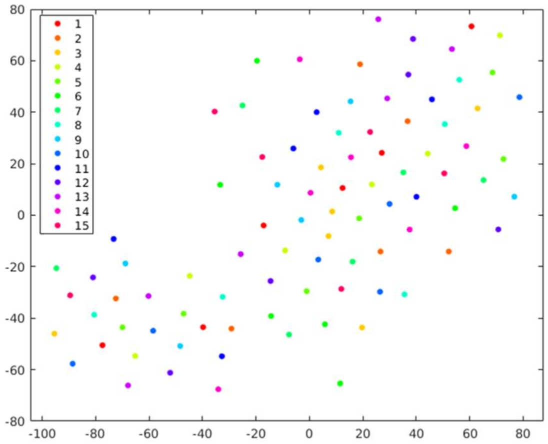

B

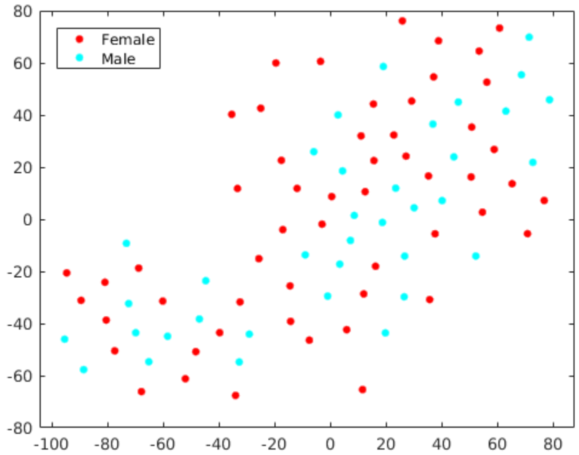

C

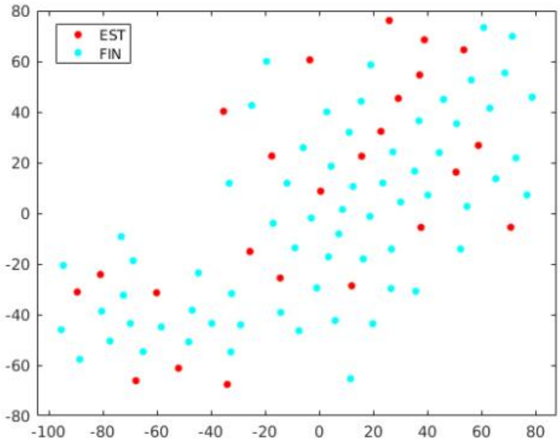

Supplementary Figure 2. Longitudinal expression profiles of six proteins measured from A) depleted serum samples collected from 15 children and analyzed with label-free quantitative profiling and B) undepleted serum samples collected from additional 8 children and analyzed with targeted SRM-based approach. Each line represents one child. Red=girls and blue = boys.

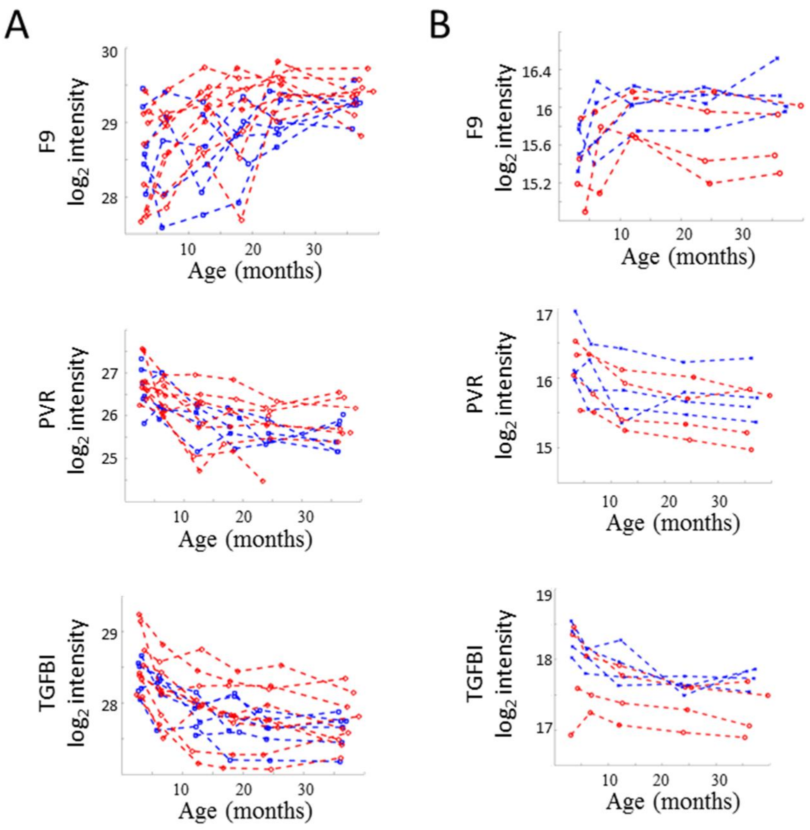

Supplementary Table 4. DAVID functional classification results for the 266 proteins reliably identified and quantified in the current study.

GO Category	Count	FDR
GO:0002576~platelet degranulation	49	3.16E-57
GO:0006508~proteolysis	48	1.52E-21
GO:0045087~innate immune response	41	4.69E-17
GO:0006956~complement activation	36	3.41E-43
GO:0006958~complement activation, classical pathway	36	7.06E-40
GO:0010951~negative regulation of endopeptidase activity	35	1.19E-30
GO:0007155~cell adhesion	35	3.69E-11
GO:0006898~receptor-mediated endocytosis	31	1.59E-20
GO:0007596~blood coagulation	30	4.95E-18
GO:0030198~extracellular matrix organization	27	4.32E-14
GO:0044267~cellular protein metabolic process	22	1.89E-13
GO:0030449~regulation of complement activation	19	1.31E-22
GO:0006953~acute-phase response	17	2.46E-15
GO:0006869~lipid transport	16	1.14E-09
GO:0042157~lipoprotein metabolic process	15	3.66E-13
GO:0007597~blood coagulation, intrinsic pathway	13	3.44E-15
GO:0042730~fibrinolysis	13	5.21E-14
GO:0006957~complement activation, alternative pathway	12	1.05E-15
GO:0034375~high-density lipoprotein particle remodeling	10	2.96E-10
GO:0043691~reverse cholesterol transport	10	2.77E-09
GO:0022617~extracellular matrix disassembly	14	1.92E-07
GO:0001895~retina homeostasis	11	6.99E-07
GO:0006955~immune response	27	2.42E-06
GO:0001523~retinoid metabolic process	12	2.95E-06
GO:0006911~phagocytosis, engulfment	10	3.20E-06
GO:0051918~negative regulation of fibrinolysis	7	3.92E-06
GO:0033344~cholesterol efflux	9	5.35E-06
GO:0050871~positive regulation of B cell activation	9	5.35E-06
GO:0017187~peptidyl-glutamic acid carboxylation	7	8.51E-06
GO:0008203~cholesterol metabolic process	12	9.86E-06
GO:0006910~phagocytosis, recognition	9	1.04E-05
GO:0001867~complement activation, lectin pathway	6	2.69E-05
GO:0019835~cytolysis	8	2.83E-05
GO:0033700~phospholipid efflux	7	5.32E-05
GO:0042158~lipoprotein biosynthetic process	6	1.57E-04
GO:0010873~positive regulation of cholesterol esterification	6	1.57E-04
GO:0050776~regulation of immune response	15	2.51E-04
GO:0006954~inflammatory response	22	7.49E-04
GO:0042742~defense response to bacterium	14	7.82E-04
GO:0042632~cholesterol homeostasis	10	8.50E-04
GO:0038096~Fc-gamma receptor signaling pathway involved in phagocytosis	12	0.00118
GO:0051384~response to glucocorticoid	11	0.001209
GO:0034384~high-density lipoprotein particle clearance	5	0.00128

GO:0030195~negative regulation of blood coagulation	6	0.001528
GO:0050853~B cell receptor signaling pathway	9	0.002462
GO:0030168~platelet activation	12	0.002653
GO:0006465~signal peptide processing	7	0.002725
GO:0034372~very-low-density lipoprotein particle remodeling	5	0.002951
GO:0070328~triglyceride homeostasis	7	0.003498
GO:0050900~leukocyte migration	12	0.004736
GO:0034380~high-density lipoprotein particle assembly	5	0.005832
GO:0031639~plasminogen activation	5	0.010371
GO:0046470~phosphatidylcholine metabolic process	5	0.017077
GO:0051919~positive regulation of fibrinolysis	4	0.022937
GO:0006641~triglyceride metabolic process	7	0.02604
GO:0034374~low-density lipoprotein particle remodeling	5	0.026513

Supplementary Table 6. Proteins whose expression levels are affected by gender, birth place and season at sampling based on GP modelling.

A. Proteins with significant gender/location effects: BestModelPosteriorRank>0.8 & scvpred.factor_vs2>0.9 & scvpred.factor_vs1>0.9.
Model 1: y ~ id, Model 2: y ~ age + id

GeneName	UniProtAccession	Variables included in the best GP model	BestModel PosteriorRank	scvpred factor vs model 2	scvpred factor vs model 1	scvpred factor 2 vs 1
ACTG1;ACTB	P63261;P60709	location,age,id	0.9069	0.92465	0.9992	0.99835
AFM	P43652	location,age,id	0.983	0.9941	0.99955	0.99545
APOC3	P02656	gender,location,age,id	0.85075	0.972	0.96615	0.72515
ATRN	O75882	location,age,id	0.89995	0.97775	0.99925	0.9958
CPB2	Q96IY4	gender,age,id	0.8462	0.9919	0.997	0.95735
CTBS	Q01459	season,id	0.8816	0.92245	0.9069	0.1431
IGFALS	P35858	gender,age,id	0.81725	0.9667	0.9839	0.78985
OLFM1	Q99784	location,age,id	0.9955	0.9984	1	0.9987
PVR	P15151	location,age,id	0.903	0.9071	0.99945	0.9983
PZP	P20742	gender,age,id	0.843	0.99115	0.96945	0.0016
SPARC	P09486	location,age,id	0.8931	0.95045	0.9698	0.9521
VWF	P04275	location,age,id	0.9078	0.9087	0.99735	0.99805

B. Suggestive GP models of longitudinal protein expression profiles (best model posterior rank between 0.5 and 0.8). Proteins with suggestive gender/location effects. BestModelPosteriorRank<=0.8 & BestModelPosteriorRank>0.5 & scvpred.factor_vs2>0.9 & scvpred.factor_vs1>0.9.
Model 1: y ~ id, Model 2: y ~ age + id

GeneName	UniProtAccession	Variables included in the best GP model	BestModel PosteriorRank	scvpred factor vs model 2	scvpred factor vs model 1	scvpred factor 2 vs 1
ACAN	P16112	location,age,id	0.56705	0.9655	1	1
AMBP	P02760	gender,location,season,age,id	0.76805	0.9576	0.97235	0.767
BTD	P43251	location,age,id	0.5676	0.9677	0.9303	0.0867
C4B	P0C0L5	gender,id	0.50775	0.9406	0.9353	0.0175
C8A	P07357	location,season,age,id	0.69385	0.97515	0.94135	0.2773
DEFA3;DEFA1	P59666;P59665	location,id	0.6768	0.9976	0.9968	0.0002
DPP4	P27487	location,age,id	0.72855	0.982	0.96275	0.351
F5	P12259	gender,season,age,id	0.67575	0.9938	0.9968	0.71385
F9	P00740	location,age,id	0.6328	0.9955	0.9977	0.9805
FN1	P02751	gender,location,season,age,id	0.58045	0.9984	0.9973	0.70395
GGH	Q92820	season,age,id	0.71535	0.9978	1	0.9989
HBB	P68871	location,age,id	0.59675	0.94985	0.9452	0.7148
HSPG2	P98160	location,season,age,id	0.5703	0.9879	0.9993	0.97265
ITIH1	P19827	location,season,age,id	0.7403	0.99925	0.9992	0.19905
LILRA3	Q8N6C8	location,age,id	0.6991	0.9732	0.94625	0.0771
LYZ	P61626	location,age,id	0.50625	0.9727	0.9715	0.7814
NCAM1	P13591	gender,location,age,id	0.6268	0.9712	0.9999	0.99975
PGLYRP2	Q96PD5	gender,age,id	0.51815	0.97025	0.99865	0.9949
PTGDS	P41222	season,age,id	0.74105	0.96445	0.9903	0.96575
SERPINA1	P01009	gender,location,age,id	0.60985	0.9262	0.90265	0.41595
TGFBI	Q15582	location,age,id	0.54995	0.99985	1	0.9903
TIMP1	TIMP1	gender,age,id	0.7459	0.92635	0.96935	0.91545
TLN1	TLN1	gender,season,id	0.5447	0.96325	0.90945	0
VTN	VTN	location,id	0.5184	0.9876	0.98895	0.6343

Supplementary Table 7. Samples and study subjects.

Study subject and samples for label-free quantitative proteomics profiling

				**Time of sample collection**						
**ChildID**	**Gender**	**Birth place**	**Mode of delivery**	**Time of Birth**	**3 months**	**6 months**	**12 months**	**18 months**	**24 months**	**36 months**
**Child1**	Female	Finland	Vaginal	Oct-2008	Feb-09	May-09	Dec-09	Apr-10	Oct-10	Nov-11
**Child2**	Male	Finland	Vaginal	Sep-2008	Dec-08	Apr-09	Oct-09	Mar-10	Aug-10	Oct-11
**Child3**	Male	Finland	Vaginal	Jan-2009	Mar-09	-	Jan-10	Aug-10	Jan-11	Jan-12
**Child4**	Male	Finland	Vaginal	Jan-2009	Apr-09	Jul-09	Jan-10	Jul-10	Jan-11	Jan-12
**Child5**	Male	Finland	Vaginal	Mar-2009	Jun-09	Sep-09	Apr-10	Aug-10	Mar-11	Mar-12
**Child6**	Female	Finland	Vaginal	Jan-2009	Apr-09	Aug-09	Feb-10	Aug-10	Jan-11	Jan-12
**Child7**	Female	Finland	Vaginal	May-2009*	Jul-09	Oct-09	May-10	Nov-10	May-11	Apr-12
**Child8**	Female	Finland	Vaginal	Jul-2009	Oct-09	Jan-10	Jun-10	Jan-11	Jun-11	Aug-12
**Child9**	Female	Finland	Vaginal	Jul-2009	Oct-09	Feb-10	Aug-10	Dec-10	Aug-11	Jul-12
**Child10**	Male	Finland	Cesarean section	Sep-2009	Dec-09	Mar-10	Oct-10	Mar-11	Oct-11	Sep-12
**Child11**	Male	Finland	Vaginal	Nov-2009	Feb-10	May-10	Nov-10	May-11	Nov-11	Nov-12
**Child12**	Female	Estonia	Vaginal	Jul-2009	Oct-09	Jan-10	Jul-10	Jan-11	Aug-11	Oct-12
**Child13**	Female	Estonia	Vaginal	Aug-2009	Nov-09	Feb-10	Sep-10	Mar-11	Oct-11	Oct-12
**Child14**	Female	Estonia	Vaginal	Feb-2010	May-10	Aug-10	Mar-11	Jul-11	Feb-12	Mar-13
**Child15**	Female	Estonia	Vaginal	Feb-2010	May-10	Sep-10	Mar-11	Aug-11	Feb-12	Mar-13

* no cord blood sample analyzed

Study subjects and samples for targeted SRM validations

				**Time of sample collection**				
**ChildID**	**Gender**	**Birth place**	**Time of Birth**	**3 months (ID*)**	**6 months (ID*)**	**12 months (ID*)**	**24 months (ID*)**	**36 months (ID*)**
**ChildA**	Male	Finland	Feb-2010	Jun-10 (26)	Aug-10 (21)	Feb-11 (18)	Feb-12 (17)	Apr-13 (2)
**ChildB**	Female	Finland	Feb-2009	May-09 (12)	Sep-09 (38)	Feb-10 (13)	Feb-11 (4)	Jan-12 (33)
**ChildC**	Male	Finland	Apr-2009	Jul-09 (1)	Oct-09 (29)	Apr-10 (15)	Apr-11 (27)	Mar-12 (20)
**ChildD**	Female	Finland	Aug-2009	Nov-09 (40)	Mar-10 (-)	Sep-10 (23)	Aug-11 (22)	Aug-12 (24)
**ChildE**	Male	Estonia	Jan-2010	May-10 (16)	Jul-10 (36)	Feb-11 (6)	Jan-12 (35)	Feb-13 (39)
**ChildF**	Female	Estonia	Feb-2009	Jul-09 (8)	Sep-09 (9)	Mar-10 (19)	Mar-11 (5)	Mar-12 (7)
**ChildG**	Male	Estonia	Jul-2009	Oct-09 (11)	Jan-10 (31)	Jun-10 (34)	Jun-11 (10)	Jul-12 (14)
**ChildH**	Female	Estonia	Jul-2009	Nov-09 (3)	Jan-10 (30)	Jul-10 (37)	Aug-11 (32)	Nov-12 (25)

* sampleID

Covariate selection for longitudinal proteomics data using additive Gaussian process regression

Lu Cheng, Aki Vehtari, Harri Lähdesmäki

May 18, 2017

Abstract

We would like to identify the most relevant factors that affect protein levels in the blood. To do this, we use Gaussian process regression to fit the proteomics time series data. In the model selection part, we use leave-one-out cross-validation and stratified cross-validation to select covariates for each protein.

1 Data description

We analyze mass-spectrometry proteomics data for a group of healthy children. The experiment is set up like this. Each child goes to the clinics at 3, 6, 12, 18, 24, 36 months age. Blood samples are taken at each visit. The samples are then analyzed together to provide the serum protein levels at those time points.

For each child, we have the following explanatory variables: age (sample date - birth date), location (Finnish or Estonian), gender, season (sample date - a common date for all child), id (id of the child).

The response variables are serum protein levels measured by mass-spectrometer and quantified by maxQuant software. The protein intensities are in log2 scale. There are 3 technical replicates for each sample. The median of the 3 technical replicates is taken as the final protein intensity. For each protein in a sample, we compute the median using only those technical replicates where the protein has been detected.

Note that many proteins can be measured in a single sample, but we analyze each protein independently. We also remove proteins that are detected in less than 50% of all available samples. In our data, we analyzed 266 proteins in total.

2 Additive Gaussian process and model selection

We describe our computational methods for a single protein by ignoring the protein index for simplicity. We can represent our data as follows: we have a continuous response variable Y and we have 5 explanatory variables X, in which 2 (age, season) are continuous explanatory variables and 3 (location, gender, id) are binary/categorical explanatory variables. We want to find out which explanatory variables can best explain the response variable.

The first question we want to answer is what modeling framework should we use. Commonly used modeling frameworks include linear mixed model and generalized linear mixed model. In practice we find linear mixed models can not fit the data very well, since the data shows nonlinear trends. We decide to use Gaussian Processes (GP) to handle the nonlinearity in the data. The general form is

$$y = f(x) + \epsilon, \tag{1}$$

where $f(x)$ can be any smooth non-linear function and ϵ is the noise.

GP is parameterized by its mean $m(x)$ and covariance matrix $k(x, x')$. In practice we usually center the data points such that the mean is 0. This practice leads to the specification of a zero-mean GP prior, which has less complex formulations while keeping the same modeling power. Assume $f(x)$ has non-zero mean μ and the noise ϵ has zero mean in Equation 1, we can rewrite it as $(y - \mu) = (f(x) - \mu) + \epsilon$, which shows the effects of centering.

The key part of GP modeling are the choices of the covariance functions. We can think the function $f(x)$ is a composed function of some more basic nonlinear functions that only involves one or two explanatory variables. Here "compose" means addition and multiplication, e.g. $f(x) = f_1(x_1) + f_2(x_2) + f_3(x_3) + f_4(x_4, x_5)$, where multiplication refers to the interaction of covariates x_4 and x_5 (or their covariance functions) in $f_4(x_4, x_5)$. Different ways of "composing" leads to different models.

Let us use M to denote a model and θ to denote parameters of the covariance functions of model M (including noise variance). We use cross-validation to compare different models. Specifically, we split the data set into small portions, then iteratively use one portion as test data and the others as training data. In the end, we would like to obtain the predictive density of one portion y_i conditioned on the rest y_{-i}, i.e. $p(y_i|y_{-i}) = \int_\theta p(y_i|\theta)p(\theta|y_{-i})d\theta$. Note that the predictive density can be obtained using sampling techniques since $p(\theta|y_{-i})$ can be sampled and $p(y_i|\theta)$ can be computed analytically. See [1] and [2] for more details.

We use two types of cross-validation in our time series data: leave-one-out cross-validation (LOOCV) and statified cross-validation (SCV). LOOCV takes out only a single data point (a single time point of an individual) and SCV takes out all data points (with respect to time) of an individual. We compare two models M_1 and M_2 by the following formula:

$$\frac{1}{n}\sum_{i=1}^{n}\left(\log(p(y_i|y_{-i}, M_1)) - \log(p(y_i|y_{-i}, M_2))\right), \tag{2}$$

which compares the average prediction accuracy of the two models. If Equation 2 is greater than 0, then M_1 is better than M_2, otherwise M_2 is better than M_1. This comparison does not provide a probability saying how much better one model compares to the other. No matter M_1 is better than M_2 at all data points, or M_1 is only marginally better than M_2 at a few data points, we will get the same conclusion, which is not favorable in many cases.

We approximate the distribution of y_i using Bayesian bootstraping [3], which assumes y_i only take values from the observations and have zero probability at all other values. The probabilities of the observation values follow the n-dimensional Dirichlet distribution $Dir(1, 1, ..., 1)$. More specifically, we bootstrap the samples

N times. Each time we get the same n observations, with each observation taking weight w_{bi} ($b = 1, \ldots, N, i = 1, \ldots, n$) from the Dirichlet distribution. Consequently, the value of Equation 2 can change for each bootstrap sample b. We then summarize the N bootstrap results to obtain the probability that M_1 is better than M_2

$$\frac{1}{N}\sum_{b=1}^{N}\delta\left\{\frac{1}{n}\sum_{i=1}^{n}w_{bi}(\log(p(y_i|y_{-i},M_1)) - \log(p(y_i|y_{-i},M_2)))\right\}, \quad (3)$$

where $\delta\{\cdot\}$ is the Heaviside step function, w_{bi} is the bootstrap weight for ith data point in bth bootstrap iteration, and weights w_b are sampled from the n-dimentional Dirichlet distribution $Dir(1, 1, ..., 1)$. See [4] for more details. We call the result of Equation 3 **loo factor** or **scv factor** depending on the cross-validation techniques used.

The above strategy also works when comparing multiple models. Instead of calculating the heaviside step function in each boostrap iteration, we simply take the model with the highest rank by sorting $\frac{1}{n}\sum_{i=1}^{n} w_{bi}\log(p(y_i|y_{-i}, M_m))$ values of all models, where m indicates the model. In the end we count the occurences N_m of each model being the best across all N bootstrap iterations and the posterior probability of model m is $\frac{N_m}{N}$, which we term as **posterior rank probability**.

3 Practical application

This section provides the technical details about how we perform the model selection for the proteomics data. In our analysis, the protein intensities and continuous covariates (age, season) are all standardized such that the mean is 0 and the standard deviation is 1. This specification allows conveniences to set the parameter priors in GP regression.

3.1 Model specification

We use GPstuff [5] to do Gaussian process regression. In our analysis, the full model space contains $2^4 = 16$ models, which are defined as follows:

$$
\begin{aligned}
&M_1 : f \sim id \\
&M_2 : f \sim age + id + age \times id \\
&M_3 : f \sim season + id + season \times id \\
&M_4 : f \sim location + id \\
&M_5 : f \sim gender + id \\
&M_6 : f \sim season + age + id + interaction\ terms \\
&M_7 : f \sim location + age + id + interaction\ terms \\
&M_8 : f \sim location + season + id + interaction\ terms \\
&M_9 : f \sim gender + age + id + interaction\ terms \\
&M_{10} : f \sim gender + season + id + interaction\ terms \\
&M_{11} : f \sim gender + location + id + gender \times location \\
&M_{12} : f \sim location + season + age + id + interaction\ terms \\
&M_{13} : f \sim gender + season + age + id + interaction\ terms \\
&M_{14} : f \sim gender + location + age + id + interaction\ terms \\
&M_{15} : f \sim gender + location + season + id + interaction\ terms \\
&M_{16} : f \sim gender + location + season + age + id + interaction\ terms,
\end{aligned}
$$

where each model uses a unique subset of covariates and the "*interaction terms*" will be explained in a later paragraph. M_1 is the constant model, which describes the protein intensity of an individual using a single constant value. M_2 is the age model, in which *age* describes the common age effect and $age \times id$ describes the individual specific age effect. Each term in a model such as *age* and $age \times id$ refers to a covariance function (kernel) used in GP regression. An interaction term is a product kernel of two kernels based on each covariate, e.g. $age \times id$ is a product kernel of the *age* kernel and the *id* kernel. Higher order interactions involving 3 or more covariates are not considered. We set the kernels and their parameter priors as follows:

1. *age* (continuous), the square exponential kernel (`gpcf_sexp()`), the length scale has the log-normal prior distribution (`prior_loggaussian()`) with $\mu = 0$ and $\sigma^2 = 1.3255$, the mode is around 0.3 and the prior penalizes small length scales; the magnitude has the log-uniform prior distribution (`prior_logunif()`), which is implemented as an improper prior in GPstuff. The posterior of the magnitude, however, is a proper distribution.

2. *location* (binary), categorical kernel times constant kernel; categorical kernel (`gpcf_cat()`) returns 1 only if two input values are the same, otherwise returns 0; prior distribution of the magnitude is a positive half Student's t-distribution (`gpcf_constant()`) with the location parameter set to 0, the scale parameter set to 1, and the degree of freedom set to 4.

3. *gender* (binary), categorical kernel times constant kernel; magnitude prior the same as for *location*.

4. *id* (categorical), categorical kernel times constant kernel; magnitude prior the same as for *location*.

5. *season* (continuous), periodic kernel `gpcf_periodic`, period = 12/(standard deviation of season covariate before standardization), length scale prior is the same as for *age*, magnitude prior is a Student's t-distribution with the location parameter set to 0, the scale parameter to 1, and the degree of freedom set to 4. Note that we impose the period to be exactly 12 months.

We assume a zero mean Gaussian noise and the prior for variance σ^2 is scaled inverse Chi-squared distribution (`prior_sinvchi2()`) with degree of freedom $\nu = 1$ and scale square (variance) $\sigma^2 = 0.1$

Regarding the interaction terms, all pair-wise interactions of the covariates in a model are included by default. We further set up and eliminate some interaction terms by the following rules:

1. Continuous covariates such as *age* and *season* are accompanied with a different length scale prior that allows for more rapid changes. For example, the length scale prior for *age* in the interaction term is the positive half of the Student's t-distribution (`prior_t()`) with the location parameter set to 0, the scale parameter set to 1, and the degree of freedom set to 4. This prior allows small length scale.

2. Interactions of continuous covariates are not considered, since they will bring too much flexiblity to the model and make the parameter inference challenging.

3. We do not consider an interaction term involving *id* and another binary covariate (*location* or *gender*) because the product kernel is in essence the same as *id* kernel, as shown in M_4, M_5 and M_{11}.

4. When considering an interaction term of a continuous covariate and a binary covariate, the constant kernel part of the binary covariate is deleted since the kernel of continuous covariate can already model the magnitude. In other words, the interaction term is a product of a squared exponential kernel and a categorical kernel.

5. When considering an interaction term of two categorical covariates, there are two constant kernels in the interaction term by default, one for each covariate. Only one constant kernel is kept due to redundency.

6. When there are more than 20 parameters in a model, we will first estimate the parameters using MCMC (see Section 3.2) and then delete interaction terms according to their explained variances. Interaction terms that explain less than 0.02 of the total variance will be deleted. If there are still more than 20 parameters, we will delete interaction terms with the least explained variances such that the final model contains 20 parameters. With more than 20 parameters, the fast central composite design (CCD) (see Section 3.2) inference algorithm is unreliable. We did not use MCMC in SCV since it was very time consuming.

We use both LOOCV and SCV to compare the aforementioned 16 models. The results show that LOOCV is good at analyzing effects of shared covariates, while SCV is good at analyzing effects of subgroup specific covariates. For example, when comparing M_4 versus M_1 using LOOCV for a protein with location effects, there is barely any difference between the two models. This is because we can borrow strength from other times points of an individual to estimate the leftout time point in LOOCV setting. If we use SCV, then we will see M_4 is much better than M_1, since M_4 uses the location mean for prediction whereas M_1 uses the whole sample mean for prediction. On the other hand, when we compare M_2 versus M_1 using SCV for a protein with age effect, the predictions will be centered around the whole sample mean, which brings in large error and can easily mask the slight improvement from *age*. As a result, there is not too much difference between M_2 and M_1. If we use LOOCV to compare M_2 and M_1, the age effect can be better detected since the baseline protein level of an individual can be reliably estimated from other time points, which circumvents the problem in SCV. We conclude that LOOCV is good at analyzing effects of covariates that are shared by all individuals, while SCV is good at analyzing effects of categorical covariates that are subgroup specific.

3.2 Inference

For each model, we use 4 independent MCMC (Markov chain Monte Carlo) chains to infer the parameters, each of which is initialized with different parameter values. Slice sampling [6] is used in the actual MCMC sampling. 2500 samples are generated from each MCMC chain, 100 samples are discarded as burn-in samples and the rest is thinned by 6, that is 400 approximately independent samples in each chain. We then concatenate the independent samples of the 4 MCMC chains, that is 1600 samples. After that we use Potential Scale Reduction Factor $\hat{R}$ [7] to check the convergence of the MCMC chains. If $0.9 \leq \hat{R} \leq 1.1$ we consider that the MCMC chains have converged. If not, we will repeat the process at most 4 times, in each of which we combine new samples with previous samples.

When performing LOOCV, we need to do the cross-validation n times such that we get predictive density of each data item, i.e. $p(y_i|y_{-i}) = \int_\theta p(y_i|\theta)p(\theta|y_{-i})d\theta$. However, this is usually time consuming and we use importance sampling to approximate the predictive density instead. Note that the leave-one-out posterior $p(\theta|y_{-i})$ is very close to the full posterior $p(\theta|y)$ since there is only one data point difference. Therefore we can use the full posterior $p(\theta|y)$ as the proposal distribution in the importance sampling, which takes little time to sample from the leave-one-out posterior $p(\theta|y_{-i})$. See more details in [1].

SCV is also time consuming if a separate MCMC inference is performed for each fold. To make the inference time affordable, we use central composite design (CCD) [8] to infer the parameters instead of MCMC. CCD assumes a unimode posterior, then it finds and assigns weights to the representative points around the posterior mode, after that it approximates the predictive density using these representative points. CCD is much faster than MCMC and reliably accurate for less than 20 parameters. Therefore we reduce the model size by removing some negligible interaction terms if a model has more than 20 parameters.

3.3 Model selection

There are two types of questions we are interested in.

1. Is there any age/seasonal effect in the data? Age/seasonal effect means that the protein intensity changes in a common pattern along time in all individuals. Since *age* and *season* are continuous covariates shared by all individuals, we use LOOCV to compare the models. The model space for LOOCV is constrained to $\{M_1, M_2, M_3, M_6\}$. The selected best model will then explain the age/seasonal effect. The following rules are adopted to select relevant models.

   (a) The best model is M_2 (`bestModelInd=2`) and its posterior rank probability $P(M_2|data)$ is higher than 0.5 (`bestModelRank>0.5`) and loo factor versus M_1 is larger than 0.95 (`loofactor_2vs1>0.95`) and MCMC converged. This rule says that the protein in question has significant age effect and no significant seasonal effect.

   (b) `bestModelInd=3 & bestModelRank>0.5 & loofactor_3vs1>0.95 & MCMC converged`. This rule says that the protein in question has significant seasonal effect and no significant age effect. There is no such protein in our result.

   (c) `bestModelInd=6 & bestModelRank>0.8 & loofactor_6vs2>0.8 & loofactor_2vs1>0.95 & MCMC converged`. This rule says that the protein in question has both significant age and seasonal effect. There is no such protein in our result.

   (d) `bestModelInd=6 & bestModelRank>0.5 & loofactor_2vs1>0.95 & MCMC converged` but does not satisfy the previous rule, i.e. `bestModelInd=6 & bestModelRank>0.8 & loofactor_6vs2>0.8 & loofactor_2vs1>0.95 & MCMC converged`. This rule says that the protein in question has significant age effect, but no significant seasonal effect.

   There does not seems to be proteins with significant seasonal effect in our result. We think this is probably because of sparse sampling in our data, i.e. 6 time points per individual is not enough to reliably detect the seasonal effect.

2. Is there any location / gender effect in the data? Location/gender effects mean that the individuals belonging to a certain group (according to location or gender) differ systematically from another group. Since gender and location are categorical covariates specific to each subgroup, we use SCV to compare all the models in the model space defined in section 3.1. If the best model (highest posterior rank probability) contains gender or location, then we think there is gender/location effect. To be considered as significant, we require that (1) the posterior rank probability is higher than 0.8 and (2) the scv factors of the best model versus both the age model M_2 and the constant model M_1 to be greater than 0.9. If the posterior rank probability is between 0.5 and 0.8, and the scv factors of the best model versus both the age model M_2 and the constant model M_1 are greater than 0.9, then we think the location / gender effect to be suggestive. Note that the SCV results can be different to that of LOOCV results.

The above filtering criteria are empirical and show satisfactory results.

References

[1] Aki Vehtari, Andrew Gelman, and Jonah Gabry. Practical Bayesian model evaluation using leave-one-out cross-validation and WAIC. *Statistics and Computing*, 2016.

[2] Aki Vehtari, Tommi Mononen, Ville Tolvanen, Tuomas Sivula, and Ole Winther. Bayesian leave-one-out cross-validation approximations for Gaussian latent variable models. *Journal of Machine Learning Research*, 2016.

[3] Donald B. Rubin. The Bayesian bootstrap. *The Annals of Statistics*, 9:130–134, 1981.

[4] Aki Vehtari and Jouko Lampinen. Bayesian model assessment and comparison using cross-validation predictive densities. *Neural Computation*, 14(10):2339–2468, 2002.

[5] Jarno Vanhatalo, Jaakko Riihimäki, Jouni Hartikainen, Pasi Jylänki, Ville Tolvanen, and Aki Vehtari. GPstuff: Bayesian modeling with Gaussian processes. *Journal of Machine Learning Research*, 14(1):1175–1179, April 2013.

[6] Radford M. Neal. Slice sampling. *The Annals of Statistics*, 31(3):705–741, 2003.

[7] Andrew Gelman, John Carlin, Hal Stern, David Dunson, Aki Vehtari, and Donald Rubin. *Bayesian Data Analysis*. Chapman and Hall/CRC, London, third edition, 2013.

[8] Håvard Rue, Sara Martino, and Nicolas Chopin. Approximate Bayesian inference for latent Gaussian models by using integrated nested Laplace approximations. *Journal of the Royal Statistical Society: Series B (Statistical Methodology)*, 71(2):319–392, April 2009.
